# Current status and perspectives of *Clonorchis sinensis* and clonorchiasis: epidemiology, pathogenesis, omics, prevention and control

**DOI:** 10.1186/s40249-016-0166-1

**Published:** 2016-07-06

**Authors:** Ze-Li Tang, Yan Huang, Xin-Bing Yu

**Affiliations:** Department of Parasitology, Zhongshan School of Medicine, Sun Yat-sen University, 74 Zhongshan 2nd Road, Guangzhou, 510080 People’s Republic of China; Key Laboratory for Tropical Diseases Control, Sun Yat-sen University, Ministry of Education, Guangzhou, 510080 People’s Republic of China

**Keywords:** Clonorchiasis, *Clonorchis sinensis*, Diagnosis, Pathogenesis, Omics, Prevention

## Abstract

Clonorchiasis, caused by *Clonorchis sinensis* (*C. sinensis*), is an important food-borne parasitic disease and one of the most common zoonoses. Currently, it is estimated that more than 200 million people are at risk of *C. sinensis* infection, and over 15 million are infected worldwide. *C. sinensis* infection is closely related to cholangiocarcinoma (CCA), fibrosis and other human hepatobiliary diseases; thus, clonorchiasis is a serious public health problem in endemic areas. This article reviews the current knowledge regarding the epidemiology, disease burden and treatment of clonorchiasis as well as summarizes the techniques for detecting *C. sinensis* infection in humans and intermediate hosts and vaccine development against clonorchiasis. Newer data regarding the pathogenesis of clonorchiasis and the genome, transcriptome and secretome of *C. sinensis* are collected, thus providing perspectives for future studies. These advances in research will aid the development of innovative strategies for the prevention and control of clonorchiasis.

**Electronic supplementary material:**

The online version of this article (doi:10.1186/s40249-016-0166-1) contains supplementary material, which is available to authorized users.

## Multilingual abstracts

Please see Additional file 1 for translations of the abstract into the five official working languages of the United Nations.

## Review

### *Clonorchis sinensis* (*C. sinensis*) and clonorchiasis

**Fig. 1:**
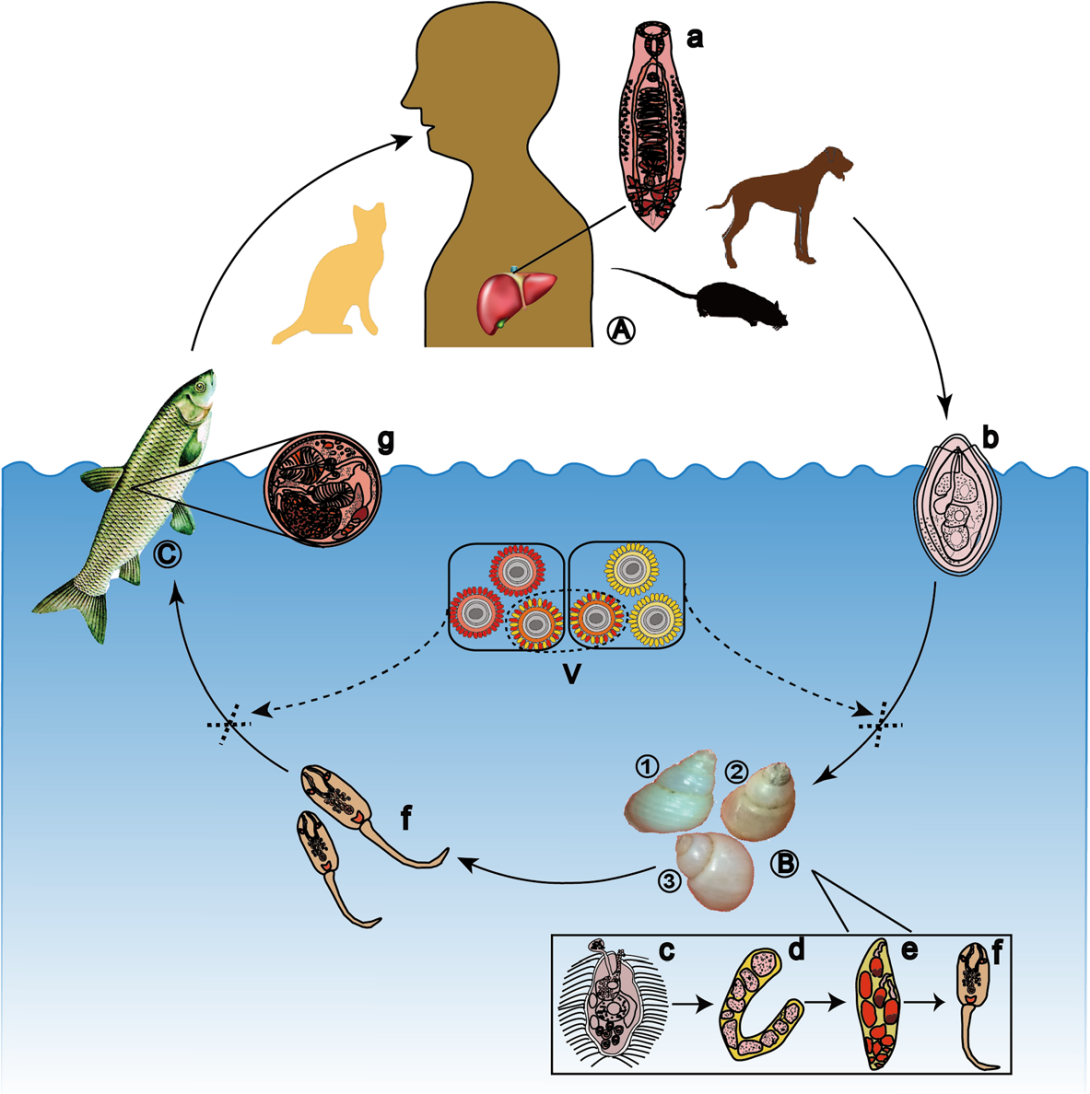
The life cycle of *C. sinensis*. The adult worms of *C. sinensis* (a) mainly live in the bile ducts of the definitive hosts (A) (human beings, dogs, cats, mice, *etc.*). The eggs (b) are discharged from the definitive hosts with the faeces. Freshwater snails, mainly *Parafossarulus striatulus* (1), *Bithynia fuchsianus* (2) and *Alocinma longicornis* (3), can serve as the first intermediate hosts (B). Eggs develop into miracidia (c) in water and then to sporocysts (d), rediae (e) and cercariae (f) after they are swallowed by snails. The mature cercariae are shed from the snails, swim freely in water, and invade into the second intermediate hosts (C) (freshwater fish) through the skin and then form metacercariae (g) in the musculature of the fish. Human or carnivorous mammals are infected due to the ingestion of raw or undercooked fish. Intervention of cercaria or metacercaria formation by effective vaccines (V) will block the transmission of *C. sinensis* and fundamentally control clonorchiasis

*C. sinensis* is a fish-borne trematode. There are three hosts in the life cycle of *C. sinensis* including freshwater snails (the first intermediate hosts), freshwater fish and occasionally shrimps (the second intermediate hosts), and human or carnivorous mammals (the definitive hosts). The life stages of *C. sinensis* include egg (in definitive hosts or water); miracidium, sporocyst, redia, and cercaria (these four stages occur in freshwater snails); metacercaria (in freshwater fish); and adult (in definitive hosts) (Fig. 1) [1, 2]. *Parafossarulus manchouricus* (*P. manchouricus*) is considered the main first intermediate host of *C. sinensis* in Korea, Russia, and Japan [3–6]. *Melanoides tuberculata* (*M. tuberculata*) serves as an important snail host of *C. sinensis* in Vietnam [7, 8]. Up to 10 species (from 3 families) of snails that are suitable for *C. sinensis* have been found in China, including *Parafossarulus striatulus* (*P. striatulus*, synonym *P. manchouricus*), *Parafossarulus sinensis*, *Bithynia fuchsianus* (*B. fuchsianus*), *Parafossarulus anomalospiralis*, *Alocinma longicornis* (*A. longicornis*), *Bithynia misella*, *Semisulcospira cancellata*, *Semisulcospira amurensis*, *M. tuberculata*, and *Assiminea lutea* [9]. Thus, a total of 10 species belonging to 3 families of freshwater snails can serve as first intermediate hosts [3–9], and most of these snails prefer places with a suitable climate and cool and slow-moving water (such as lakes, streams, ponds, marshes, paddy fields and small ditches). *P. striatulus*, *A. longicornis* and *B. fuchsianus* are the main freshwater snails that can be infected. *Pseudorasbora parva* (*P. parva*) is the most important second intermediate host of *C. sinensis*, followed by other freshwater fish, such as *Ctenopharyngodon idellus* (*C. idellus*), *Carassius auratus* (*C. auratus*), *Cyprinus carpio* (*C. carpio*), *Hypophthalmichthys nobilis* (*H. nobilis*) and *Saurogobio dabryi* of Cyprinidae [1, 10]. In the Republic of Korea, approximately 40 species of freshwater fish (31 genera in 6 families) are suitable as second intermediate hosts of *C. sinensis* [11]. In China (Taiwan included), a total of 102 species of fish (59 genera in 15 families) and four species of shrimp are recognized as hosts [12]. Seven species of fish are infected by *C. sinensis* metacercariae in the Amur River of Russia [6]. In addition to humans, cats, dogs, and other carnivorous mammals can serve as natural reservoir hosts of *C. sinensis*. In addition, rats, hamsters, rabbits and mice are usually used to prepare experimental animal models of *C. sinensis* [2, 9, 10].

The final hosts are infected with *C. sinensis* through the ingestion of raw or undercooked freshwater fish containing metacercariae. Metacercariae excyst in the duodenum of the host before moving to the bile ducts and further developing into adults [1, 13]. The adult worms can survive for a long period in the bile ducts, causing clonorchiasis in humans. Clonorchiasis generally appears as jaundice, indigestion, biliary inflammation, bile duct obstruction, even liver cirrhosis, cholangiocarcinoma (CCA), and hepatic carcinoma [13, 14]. Almost 5 000 CCA cases attributed to *C. sinensis* infection may occur annually in the coming decades in East Asia [15]. *C. sinensis* is classified as a group I biocarcinogen by the International Agency for Research on Cancer (IARC) of the World Health Organization (WHO) [16].

### Prevalence and disease burden of clonorchiasis

Clonorchiasis is mainly prevalent in Asian countries and regions, including South Korea, China, northern Vietnam, and far-eastern Russia [1, 14, 17]. Moreover, emigrants or travellers from endemic areas will increase the risk of disease transmission to other countries [17]. Currently, it is estimated that more than 200 million people are at risk of infection with *C. sinensis* worldwide, over 15 million people are infected, and 1.5–2 million people show symptoms or complications [13, 15, 18]. China has the largest population of infected people, which is estimated at 13 million [9, 17, 19].

Two nationwide surveys (in 1989–1992 and 2001–2004) on important parasitic diseases of humans (clonorchiasis included) have been carried out in China [19–21]. Average *C. sinensis* infection rates found in the first and second national surveys were 0.31 % and 0.58 %, respectively [19, 21]. In the 2001–2004 survey, an epidemiological investigation of clonorchiasis was conducted in a total of 27 endemic provinces/autonomous regions/municipalities (P/A/M). *C. sinensis* infection was found in a total of 19 P/A/M, and the average infection rate among the 27 P/A/M was as high as 2.4 % [19, 20]. It is estimated that 12.49 million individuals are infected with *C. sinensis* in mainland China; the infection rate in Guangdong province is the highest (16.4 %), followed by those in Guangxi autonomous region (9.8 %) and Heilongjiang province (4.7 %) [19, 20].

The prevalence of *C. sinensis* in intermediate and reservoir hosts in China during the last decade is presented in Additional file 2: Tables S1-S3. *P. striatulus*, *A. longicornis* and *B. fuchsianus* remained the main carriers of *C. sinensis* cercariae, and the positive rates for these were 0.13–17.4 %, 0–7.4 % and 0–1.28 %, respectively (Additional file 2: Table S1).

Almost 31 types of freshwater fish/shrimp were reported to be infected with metacercariae of *C. sinensis* in 14 P/A/M of China, most of which were members of Cyprinidae (Additional file 2: Table S2). Common edible fishes (e.g., *C. idellus*, *C. auratus, H. nobilis*, *C. carpio*, *H. molitrix* and *M. anguillicaudatus*) collected from markets, restaurants, fishponds and rivers continued to have a high probability of carrying the metacercariae of *C. sinensis*. Positive rates for *C. idellus*, *H. nobilis* and *C. carpio* remained high in most areas of China, especially in southern regions, such as Guangdong, Guangxi and Hunan. Moreover, the rate of infection with *C. auratus* was relatively high in Heilongjiang and Liaoning provinces. Small fish, such as *P. parva*, *Cyprinidae Rhodeus*, *Abbottina rivularis* and *Hemicculter Leuciclus* are distributed widely and are highly susceptible to infection (up to 100 %) (Additional file 2: Table S2).

The existence of reservoir hosts is closely related to the maintenance and development of the life cycle of *C. sinensis*. As shown in Additional file 2: Table S3, the prevalence of *C. sinensis* in cats and dogs was high in Guangdong, Guangxi, Heilongjiang and Jiangsu provinces, followed by Hunan, Hubei, Shandong and Liaoning. The rate of infection in cats was higher than that in dogs, and pigs were infected at lower levels. Rabbits were documented to be infected with *C. sinensis* in Heilongjiang province. Other animals, such as cattle, ducks, voles, chicken, and *Rattus norvegicus* were not found to be infected (Additional file 2: Table S3). Therefore, importance should be attached to the prevention and control of reservoir host infection by feeding pets with cooked or specially processed food and improving the management of pet faeces.

In South Korea, the prevalence of egg-positive individuals was 4.6 % in 1971, 1.8 % in 1976, 2.6 % in 1981, 2.7 % in 1986, 2.2 % in 1992, 1.4 % in 1997 and 2.9 % in 2004. It is estimated that 1.4 million people are currently infected with this fluke. People near the Nakdong River showed the highest egg-positive rate (40–48 %) [22].

Clonorchiasis has been recorded in almost all northern provinces of Vietnam at prevalence values ranging from 0.2 to 37.5 %; the rates are especially high in the Red River delta region. The highest infection rate (26.0–37.5 %) is found in Nam Dinh province, followed by Ninh Binh province (23.5–31.0 %) [23, 24]. In Russia, *C. sinensis* is mainly distributed in the southern Far East, especially near the Amur River basin, and approximately 3 000 people are estimated to be infected [15, 17, 25].

The infection rate is generally higher in males than in females. People who habitually eat raw or undercooked fish and eat outside frequently have higher infection rates than those who do not. *C. sinensis* infection is most serious in elderly people aged 40–60. Businessmen, fishermen, workers, farmers and catering staff exhibit higher infection rates [19–21].

Most cases of clonorchiasis occur in low- or middle-income countries of Asia, causing severe disease burdens and serious medical and economic problems. It is estimated that the global burden of clonorchiasis is approximately 275 370 disability-adjusted life years (DALYs), and almost 5 591 people die from the infection each year [18]. The calculated economic burden of clonorchiasis-induced cholecystitis, gallstones, liver cirrhosis, and liver cancer in Guangdong province (China) was RMB 1.3 billion (more than $US 200 million) [26]. Thus far, no national investigation of the economic burden has been conducted in China or in other endemic areas. It has been documented that overall disability is higher in males than in females, and disability and infection intensity are positively correlated (gallstones are responsible for the greatest proportion) [27].

### Diagnosis and treatment

Adult worms of *C. sinensis* can inhabit bile ducts for 20–25 years, and there are no obvious clinical symptoms at the early stage of the infection, often resulting in missed diagnosis [1]. In addition, clonorchiasis is usually misdiagnosed due to its nonspecific symptoms, such as fatigue, inappetence, nausea, bellyache, jaundice, and hepatosplenomegaly [15]. People who live in or have come from epidemic areas, have consumed raw or undercooked freshwater fish, and appear with the above symptoms should be considered suspect for clonorchiasis.

Eggs found in stool can confirm *C. sinensis* infection. Stool examination is inexpensive and does not require the use of sophisticated equipment; however, labour intensive, inconvenient, and well-trained staff are needed [1, 13]. Direct faecal smear, the Kato-Katz (KK) method and the formalin-ether concentration technique (FECT) are commonly used stool examination methods for diagnosis [28, 29]. Hong et al. reported that although FECT was more sensitive than the KK method for diagnosing very light infection cases, the KK method was more reliable for diagnosing clonorchiasis [29]. Another study demonstrated that the KK method and direct smear were suitable for the large-scale epidemiological screening of clonorchiasis [28]. Qian et al. proposed that the KK method was more reliable than FECT for the diagnosis and drug efficacy evaluation of clonorchiasis [30]. However, the eggs of *C. sinensis* are easily confused with the eggs of other flukes (e.g., *Opisthorchiidae*, *Lecithodendriidae* or *Heterophyidae*) [1, 13].

Serological methods are helpful for the diagnosis of clonorchiasis. A specific antibody or antigen of *C. sinensis* can theoretically be detected in serum samples. The detection of a specific antibody is more often applied than the detection of an antigen due to the trace amount of antigen present and sensitivity limitations, although Nie et al. showed that an IgY (egg yolk immunoglobulin)-based immunomagnetic bead enzyme linked immunosorbent assay (ELISA) system (IgY-IMB-ELISA) appears to be a sensitive and specific assay for the detection of circulating antigen in human clonorchiasis and a significant correlation has been found between ELISA optical density and egg counts (EPG) [31]. Crude extracts of *C. sinensis* are considered sufficiently sensitive for the serodiagnosis of clonorchiasis, but cross-react with other trematodes [13, 32]. Regarding the source and standardization of crude extracts, many purified recombinant proteins from the tegument or excretory/secretory proteins (ESPs) of the worm such as the 21.1-kDa tegumental protein, cathepsin L proteinase, cysteine protease, and a fragment of paramyosin (*Cs*PmyC-2) [33–36] have been evaluated regarding their potential role in diagnosis. Compared with IgG-ELISA or IgG4-ELISA, the IgG4-ABC (avidin-biotin complex)-ELISA that detects a specific IgG4 antibody against *C. sinensis* is more sensitive and specific [37]. Until now, two diagnostic kits (No. 3400107 and No. 3401500) that detect specific antibodies in an ELISA system have been authorized by the Chinese Food and Drug Administration (CFDA).

DNA-based methods can also be used for diagnosis. A variety of PCR techniques (including conventional PCR, real-time PCR, multiplex PCR, PCR-RFLP and FRET- PCR) have been employed. Sensitive and specific target genes, such as internal transcribed spacers (ITS-1 or ITS-2) of nuclear ribosomal DNA (rDNA), cox1, Rn1 and nad2 have been documented [13, 38–40]. However, special equipments are required for PCR; therefore, these techniques are not suitable for use in less developed areas. Loop-mediated isothermal amplification (LAMP) is confirmed as being more sensitive, rapid and applicable in a resource-poor setting than PCR. LAMP has been successfully applied for the detection of human *Opisthorchis viverrini* (*O. viverrini*), *Paragonimus westermani*, *Fasciola hepatica*, and *Fasciola gigantica*, among others [41–43]; however, no effort has been made to develop LAMP or any other isothermal approaches for human clonorchiasis diagnosis.

Imaging methods including ultrasound, computer tomography (CT), magnetic resonance imaging (MRI) and tissue harmonic imaging (THI) have important accessory diagnostic values and are also employed to assess disease progression; however, these methods exhibit relatively poor sensitivity and are nonspecific, and it can be difficult for inexperienced staff to use these methods. In addition, these techniques can be expensive to employ [1].

Clonorchiasis can be treated effectively with praziquantel (PZQ) on early accurate diagnosis and correct species identification [44]. According to WHO recommendations, treatment with doses of 25 mg/kg thrice daily for two consecutive days can achieve cure rates of 93.9–100 % [25, 45, 46]. Choi et al. conducted a clonorchiasis control project in endemic areas in China during 2001–2004 and found that repeated mass treatment or selective treatment with PZQ every 6 to 12 months was highly effective (yielding low prevalence and re-infection rates, and a high egg reduction rate) for clonorchiasis control in heavily endemic areas; in moderately endemic areas, only 1–2 selective treatments were required when combined with health education [47]. A programme involving repeated PZQ treatment at 6-month intervals was carried out in an endemic village in Korea. The egg-positive rate decreased from 22.7 % (in 1994) to 6.3 % (in 1998), but the treatment was insufficient to achieve the complete control of clonorchiasis [48]. Occasionally, the efficacy of PZQ against clonorchiasis is poor and fails to achieve a satisfactory effect despite long-term repeated therapy [49]. In 1997, a pilot study conducted in northern Vietnam only reached a 29 % cure rate after treating clonorchiasis patients with 25 mg/kg PZQ once daily for 3 days; perhaps this dosage of PZQ is inappropriate [49]. In addition, mild and transient adverse events including dizziness, headache, vomiting, sleepiness, diarrhoea, headache, and allergy may occur after taking PZQ [17, 47, 50]. Thus, a wider choice of drugs for the treatment of clonorchiasis should be developed. Tribendimidine has proven highly effective for treating *C. sinensis* in vitro and in rats and hamsters [51, 52]. Two recent comparative clinical studies demonstrated that compared to PZQ, tribendimidine is as efficacious for the treatment of *C. sinensis* infection and in some dosage regimens is even more convenient and effective (Table 1) [53–57]. Other drugs such as artemether, artesunate, OZ78 and mebendazole have also been evaluated for treating *C. sinensis* infection in animal models [58, 59].

**Table 1:** Therapeutic schemes and drugs for clonorchiasis

Therapeutic regimen		Disease information	Treatment effect	References
Praziquantel	Orally, 18.8 mg/kg twice daily for 2 days.	Co-infection with other helminthes.	CR: 56.8 % (1st, 21/37), 75 % (2nd, 12/16).	[53]
	Orally, 25 mg/kg three times for 1 day.	No other illness.	CR: 56 % (14/25).	[54]
	Orally, 25 mg/kg for three times.	No other illness.	CR: 62.9 %(83/132),	[55]
	Orally, 3.6 g/d three times daily for 2 days, combined with ENBD.	Severe infection, jaundice.	Effective and safe.	[56]
SRP	Orally, 30 mg/kg once.	Severe infection.	CR: 60 % (12/20), side effects.	[57]
Tribendimidine	Orally, 400 mg once.	Co-infection with other helminthes.	CR: 50 % (1st, 17/34), 78.1 % (2nd, 25/32).	[53]
	Orally, 200 mg twice for 1 day.	Co-infection with other helminthes.	CR: 33.3 % (11/33).	[53]
	Orally, 400 mg once.	No other illness.	CR: 44 % (11/25).	[54]
Mebendazole	Orally, 400 mg once.	Co-infection with other helminthes.	CR: 0 % (0/30)	[53]

*CR* cure rate, *ENBD* endoscopic nasobiliary drainage, *SRP* sustained-releasing praziquantel, *1st* the first treatment, *2nd* the second treatment

### Vaccine development

No commercially produced or effective vaccine is available for the treatment of *C. sinensis* infection in human or other hosts as of yet. Researchers have obtained some protective effects, but only in rat models [60–68]. Quan et al. reported that rats that were pretreated with irradiated metacercariae of *C. sinensis* at the single dose of 12 Gy can generate resistance to infection, as characterized by low worm recovery, high IgG antibody titre and high levels of IFN-γ and IL-2 [60].

The key molecules in the life cycle of *C. sinensis* (including components of ESPs, tegumental proteins, and metabolism-related enzymes) have been identified as potential vaccine candidates [69]. Worm reduction rates of 31.50, 40.90, 31.60 and 37.42 % were elicited by the intramuscular injection of a plasmid containing genes encoding cysteine proteinase, fatty acid-binding protein, *Cs*PMY and enolase (*Cs*ENO), respectively [13, 61, 62]. Furthermore, worm reduction rates of 60.4, 45.38, 54.30, 41.00, 67.00, 56.29 and 50.20 % were elicited by subcutaneous inoculation with the recombinant proteins Rho GTPase, 14-3-3 epsilon, *Cs*PMY, cathepsin B cysteine protease 2 (*Cs*CB2), *Cs*CB3, *Cs*ENO and hexokinase (*Cs*HK), respectively [62–66]. Finally, worm reduction rates of 44.70 and 60.07 % were elicited in rats by the oral delivery of *Bacillus subtilis* spores expressing a 22.3 kDa tegumental protein of *C. sinensis* and *Cs*ENO, respectively [62, 67, 68].

### Pathogenic mechanisms of clonorchiasis

The fact that the pathogenesis of clonorchiasis (especially liver fibrosis and CCA induced by the infection of *C. sinensis*) remains unclear has slowed the development of effective prevention and control strategies.

Periductal fibroplasia induced by *C. sinensis* occurs at an early stage of the infection (e.g., 7 days after infection); fibroplasia then develops into liver parenchyma (our unpublished data). The progress of this disease is different from that of the hepatic fibroplasia that results from the hepatitis induced by hepatic virus and alcohol. The molecular mechanisms that are involved in these diseases are thought to be different. ESPs from *C. sinensis* have been shown to play roles in the progress of this disease. As components of ESPs, secretory phospholipase A (2) (*Cs*PLA2), lysophospholipase (*Cs*lysoPLA), fructose-1, 6-bisphosphatase (*Cs*FBPase) and Fe heavy chain protein (*Cs*FHC) have been reported to directly activate human hepatic stellate cells (HSCs) and key cells in liver fibrosis and to prompt the production of collagen [70–74]. The TGF-β/Smad signalling pathway might be activated after infection, which might contribute to the synthesis of collagen type I and fibroplasia [75]. Zheng et al. intraperitoneally injected *C. sinensis* calmodulin (*Cs*CaM) into rats and observed severe liver inflammation with mild to moderate liver fibrosis as the result [76].

A study of the cancer-critical genes of *C. sinensis*-associated CCA showed that *PSMD10* and *CDK4* genes were upregulated, the tumour suppressor gene *p53* and RB protein as well as *BAX* and *caspase 9* were down-regulated, and PCNA was overexpressed in a *C. sinensis-*induced hamster CCA model [77]. Several researchers have proposed that oxidative stress might mediate liver fluke-associated carcinogenesis [78–80]. Serious pathological changes and increased DNA lesion products (resulting in the accumulation of lipid peroxidation products and the activation of COX-2 and 5-LOX) occurred in the hepatobiliary system of *C. sinensis-*infected mice, accompanied by the obvious activation of inducible NOS (iNOS) and malondialdehyde (MDA) [78–80]. The NOS-interacting protein of *C. sinensis* (*Cs*NOSIP) might be a key participant in the oxidative stress observed in clonorchiasis [80].

Infection with *C. sinensis* might induce the hydropic degeneration of hepatocytes through the Fas/FasL-mediated pathway. ESPs and the components of ESPs (e.g., *Cs*severin) might suppress the apoptosis of malignant/abnormal cells (HuCCT1, a CCA cell line; or PLC, a hepatocarcinoma cell line), possibly inducing the development of tumours [13, 81, 82]. Kim et al. demonstrated that ESPs of *C. sinensis* could increase the proliferation of HuCCT1 cells (a human epithelial cell line) by reducing parthenolide-induced apoptosis [82]. In another study, after treating HEK293 cells (a human epithelial cell line) with ESPs plus the carcinogen dimethylnitrosamine, the proportion of cells in the G2/M phase and the expression of cell cycle proteins (e.g., E2F1, p-pRb and cyclin B) were markedly enhanced [83]. Using microarrays, Kim et al. studied gene expression profiles in ESP-treated HuCCT1 cells, and a total of 23 920 genes were found to be differently expressed. Among the up-regulated genes, minichromosome maintenance protein 7 was implicated in various cancer types [84]. Evidence was obtained that ESPs can promote the three-dimensional aggregation and invasion of HuCCT1 into the neighbouring extracellular matrix due to the expression of focal and cell-cell adhesion proteins and the secretion of matrix metalloproteinases [85]. Another study identified 16 dysregulated miRNAs (13 were up-regulated, and 3 were down-regulated) including the decreased expression of let-7i (a tumour suppressor miRNA) when HuCCT1 cells were treated with ESPs for different times [86].

In addition, immune responses such as inflammation are proven to be involved in the pathogenesis of fibrosis and carcinoma. Nam et al. revealed that free radicals that are enzymatically triggered by *C. sinensis* ESPs can cause NF-kB-mediated inflammation in HuCCT1 cells [86]. Chronic inflammation can damage DNA and might result in the malignant transformation of cells. Crude antigens of *C. sinensis* and ESP components (e.g., *Cs*RNASET2) might markedly elevate Th2-associated cytokines such as IL-4, IL-5 and IL-13, IL-10 and TGF-β in *C. sinensis*-infected mice through their action on dendritic cells [87–90]. Additionally, levels of IL-33/ST2 (a potent inducer of bile duct proliferation and fibrosis) were highly increased in *C. sinensis*-infected patients and mice, and the Treg/Th17 ratio was also increased in *C. sinensis*-infected mice [91, 92]. Moreover, the chemokines RANTES and MIP-1α were also upregulated [87]. TGF-β, IL-13 and IL-10 are well-known cytokines that can activate HSC to produce collagen types I and III (our unpublished data). These cytokines also act as anti-inflammatory agents, possibly helping the worm to evade the immune response, enabling it to survive in bile ducts for a long time and finally causing long-term chronic inflammation. TLR2 and TLR4 were reported to be upregulated in a mouse model of clonorchiasis for defence against *C. sinensis* infection and pathogenicity. High TLR4 expression induced the secretion of pro-inflammatory cytokines (TNF-α and IFN-γ) in ESP-stimulated biliary epithelial cells [93, 94]. In our opinion, *C. sinensis*-induced liver fibrosis is a mechanism that protects the host and represents an immune pathological phenomenon, and CCA results from an imbalance between inflammation and repair.

In summary, the precise mechanisms should be further explored such that the progress of fibrosis and carcinoma can be stopped by interfering with the corresponding pathways or molecules.

### The genome, transcriptome and secretome of *C. sinensis*

Omics, including genomics, transcriptomics and proteomics can help us to learn more regarding the migration, parasitism and pathogenesis of *C. sinensis* at the molecular levels, which would be extremely helpful for the development of new and effective prevention and control strategies against clonorchiasis.

The whole-genome size of *C. sinensis* was assumed to be 580 Mb, and the GC content was calculated as approximately 43.85 %. Heterozygosity was approximately 0.4 % for the entire genome. In *C. sinensis*, approximately 32 % of the genome constitutes interspersed repeats based on known and *ab initio* repeat libraries. A total of 13 634 gene models were identified. Genes for the complete pathways of glycolysis, the Krebs cycle and fatty acid metabolism were found. Nearly 60 % (2 203/3 675) of *C. sinensis* protein domains are shared with other taxa [69, 95]. Complementary DNA libraries were constructed from the adult, metacercaria, and egg of *C. sinensis* to obtain the gene transcript of the worm. A total of 52 745 expressed sequence tags (ESTs) were generated and assembled into 12 830 *C. sinensis* EST sequences. Energy metabolism, protease, antioxidant enzyme, motility and reproduction genes were differentially expressed in adults. Minimal metabolism and host adaptation genes were differentially expressed in metacercariae, and embryonic genes were differentially expressed in eggs [96]. Moreover, to discover new transcribed isoforms and to comprehensively characterize gene expression dynamics among different tissues, RNA-Seqs of oral sucker, muscle, ovary and testis tissues of *C. sinensis* were performed. Approximately 26 % (3 535/13 634) of the gene models had two or more transcribed isoforms. In total, 14 087 alternative-splicing events grouped into 11 different splicing patterns were detected. In addition, 4 259 transcribed regions corresponding to 4 821 transcripts were newly identified. In total, 9 860 genes and 19 435 transcripts were expressed in at least one tissue, and 9 459 genes (69.4 %) were expressed in all four tissues from the adult worm. Differently expressing genes (DEGs) were identified: 1 094 in muscle vs. oral sucker, 1 315 in muscle vs. ovary, 1 043 in muscle vs. testis and 516 in ovary vs. testis. Genes that were expressed at high levels in the testis were enriched in microtubule-based movement, microtubule-based processes, negative regulation of actin filament polymerization and negative regulation of protein polymerization categories. Genes that were highly expressed in both testis and ovary were enriched in spermatogenesis, sperm motility, and male gamete generation and fertilization pathways. Genes that were highly expressed in the oral sucker were enriched in pathways related to lipid binding, stimulus response and muscle differentiation, and genes that were highly expressed in muscle were enriched in pathways that are related to metabolic function [95].

The genome and transcriptome data demonstrated that the glycolysis, TCA cycle and oxidative phosphorylation pathways were similar to those for two other parasites, *Schistosoma mansoni* in blood and *Ascaris suum* in the intestine, but the pattern of fatty acid-related gene expression in *C. sinensis* was different [95].

Based on the genome and transcriptome data, non-coding RNA genes were identified. rRNA, tRNA, snoRNA, snRNA and miRNA were 0.0006, 0.0037, 0.0065, 0.0022 and 0.0032 % of the genome, respectively [95]. Xu et al. identified and cloned 6 new and 62 512 conserved adult *C. sinensis* miRNAs which are grouped into 284 families [97]. In another study, 33 novel and 18 conserved miRNAs were identified in *C. sinensis* (adult worms), including csi-miR-36b, which was not found by Xu et al. [97, 98]. The miRNAs of *C. sinensis* were concentrated along three branches of the phylogenetic tree leading to bilaterians, insects and coelomates. In total, 256 990 microsatellites have been identified in the whole genome of *C. sinensis* through the SciRoKo programme. The ATC repeat is the most abundant microsatellite, and 24 microsatellite markers show potential application in the study of genetic diversity [99].

The ESPs of parasites play important roles in host-parasite interactions; therefore, the components and functions of ESPs have attracted the interest of researchers. Ju et al. isolated *C. sinensis* adults from experimentally infected rabbits and incubated them with PBS for 4 h [100]. Sixty-two protein spots were identified in the concentrated supernatant using 2-DE-based mass analysis and the EST database of *C. sinensis*. Of these, detoxification enzymes, such as glutathione S-transferase and thioredoxin peroxidase, myoglobin and a number of cysteine proteases were expressed abundantly. In another study, *C. sinensis* adults were collected from naturally infected cats and cultured in DMEM from 0 to 48 h at intervals of 12 h. The supernatant containing ESP was dialysed against PBS and further analysed. In total, 110 proteins including 71 hypothetical proteins of unknown function and 39 proteins of various functional categories were identified by shotgun LC–MS/MS. The 39 proteins could be classified as glycometabolic enzymes, detoxification enzymes, structural proteins and several RAB family proteins [101]. Furthermore, ESPs from *C. sinensis* adults cultured in DMEM for different periods (0–3 h, 3–6 h, 6–12 h, 12–24 h, 24–36 h, and 36–48 h) were also analysed, and 187, 80, 103, 58, 248, and 383 proteins were found, respectively. Twenty-four proteins of known function and other hypothetical proteins were detected. The 24 proteins were grouped into various functional categories: ribosome proteins, enzymes, enzyme inhibitors and other proteins [102]. These studies showed that the components of ESPs might differ between definitive hosts as well as when cultured for different periods in vitro. More omics information relating to *C. sinensis* are shown in Table 2 [69, 95, 97–99, 103–106].

**Table 2:** Omics information of *C. sinensis*

Genome	Draft genome 580 Mb, GC content 43.85 %, 13 634 gene models, mitochondrial genome 13 875-13 879 bp, 256 990 microsatellites [69, 95, 99, 103–105].
Transcriptome	27 082 transcripts, 88 714 ORFs, 39 novel and 65 530 conserved miRNAs in adult worms, genes differentially express in different stages or tissues [95, 97, 98, 106].
Proteome	50 769 protein domains, participating in diverse biological processes [69, 95, 106].

### Prevention and control strategy

The prevention and control strategy of clonorchiasis usually involves a combination of two or more measures, including health education, health promotion, chemotherapy and environmental reconstruction [107]. The strategies proposed by the Centers for Disease Control and Prevention (CDC) of the USA, WHO, and the National Health and Family Planning Commission of the People’s Republic of China (NHFPC) are summarized in Table 3 [108–110].

**Table 3:** Prevention and control strategies of clonorchiasis proposed by CDC, WHO and NHFPC

CDC	Do not eat raw or undercooked freshwater fish, cooking fish adequately (internal temperature > 63 °C), freezing (≤ −20 °C for 7 days; ≤ −35 °C for 15 h).	http://www.cdc.gov/parasites/clonorchis/faqs.html [108]
WHO	Recommending veterinary public health measures and food safety practices to reduce the risk of infection, improving safe and effective of anthelminthic medicines to control morbidity.	http://www.who.int/mediacentre/factsheets/fs368/en/ [109]
NHFPC	Improving the coverage of norms anthelmintic drugs and the sanitary toilets; increasing the rates of knowledge about prevention and control of parasitic and health behavior, improving the qualified rate of medical personnel in the township (town) or village.	http://www.nhfpc.gov.cn/zhuzhan/zcjd/201304/cba68ffe544c4902bd48b1cd7d41e733.shtml [110]

Health education includes the broadcast of educational programmes on television, broadcasts and VCDs, billboard/propaganda painting, the distribution of health guide booklets, and the transmission of disease-related knowledge to residents and school children [107, 111, 112]. Removing toilets and pigsties from fishpond areas is helpful in terms of environmental reconstruction. Health education and promotion programmes can enhance knowledge regarding *C. sinensis* infection and the need to avoid the consumption of raw or undercooked freshwater fish, which would generally promote the process of chemotherapy and environmental reconstruction.

Mass chemotherapy using PZQ has been adopted in many endemic areas and shows promise regarding the successful control of clonorchiasis [47]. Thus far, chemotherapy in combination with health education is more effective and provides a more long-lasting control than the use of chemotherapy alone [47, 107]. Considering the potential tolerance and side effects of PZQ, the development and promotion of safe and effective anthelminthic drugs have been proposed. However, high infection rates persist in endemic areas due to the lack of culturally sensitive and educationally informed information concerning ‘raw attitudes’ in the eating patterns of the population [23].

Removing exposure to a raw freshwater fish/shrimp diet might be the most effective way to block infection by *C. sinensis* as well as other fish-borne zoonotic trematodes [113]. Residents in the epidemic area find it difficult to change their habit of eating raw fish/shrimp; moreover, they have more opportunities to ingest food containing raw fish. Therefore, more attention should be paid to the safety of freshwater fish. The infection rates and distribution of freshwater fish and snails should be investigated in endemic areas, and infected ponds should be placed under surveillance. Additionally, metacercaria-tainted fish should be barred from markets.

The direct compression or artificial digestion of fish followed by detection under a microscope is used to examine *C. sinensis* metacercariae in freshwater fish. This method is time consuming, labour-intensive, and *C. sinensis* is easily confused with other parasites (e.g., *Opisthorchiidae*, *Heterophyidae* and *Lecithodendriidae*) at this stage [113, 114]; thus, rapid, convenient and accurate detection methods are urgently needed. PCR-based molecular biology techniques including nested PCR, real-time PCR, multiplex PCR and a LAMP have been employed to detect the infection of fish and snails in recent years [113–119]. ITS-1 and ITS-2 rDNAs of *C. sinensis* are the main target genes used [113–115, 117–119]. Cai et al. developed a TaqMan-based real-time PCR assay for the detection of *C. sinensis* DNA in fish; amounts as low as 1 pg of purified genomic DNA and one metacercaria per gram of fish filet can be detected [119]. Additionally, metacercariae of *C. sinensis* and *O. viverrini* in fish can be discriminated using PCR-based techniques. Chen et al. and Cai et al. reported that the LAMP assay was 100 ~ 1 000 times more sensitive than conventional PCR for the detection of *C. sinensis* metacercariae and was more suitable for use in the field [113, 120]. Due to their well-developed immune system, bony fish can generate specific anti-heterogeneous antigens IgM in their blood and mucus. Immunological detection is also a promising method for the rapid and accurate detection of infection in fish.

The infection rates of clonorchiasis in humans are generally and positively correlated with those in animals. High infection rates in people can usually aggravate the reservoir hosts’ infection. In contrast, animals (e.g., cats and dogs) that defecate widely, accelerate the transfer of eggs into ponds and rivers, thus promoting the completion of the parasite life cycle and aggravating the epidemic [10, 121]. Infected carnivorous mammals remain large threats even if human beings have been treated. These animals are infected by the ingestion of metacercaria-containing fish. Controlling this process is an ideal way to protect fish from infection and to reduce the transmission of *C. sinensis* (Fig. 1). Using non-polluted water for the culture of fish combined with the use of a fish vaccine against *C. sinensis* infection will be helpful to reduce *C. sinensis* infections and aid in the supervision of the safety of freshwater fish.

### Research priorities for blocking transmission of the disease

Currently, many difficulties and gaps remain in the study of blocking the transmission of this disease. First, the rate and degree of infection of the human and animal populations, and the distribution of endemic areas (e.g., in counties, towns or villages) are not clearly known or understood in real-time. The phenomena of missed diagnosis and misdiagnosis remain serious [15, 122] and delay the treatment of patients and reservoir hosts. The factors are the main difficulties in controlling clonorchiasis. The examination of eggs remains the gold standard for diagnosing clonorchiasis [13, 123]; however, technologies that yield rapid, convenient and accurate diagnosis are urgently required for large-scale screening and clinical application.

Second, although some researchers have obtained information regarding the developmental cycle of the parasite in snails or fish infected with *C. sinensis* under laboratory conditions [124], insufficient knowledge of the living habits, life characteristics and infection mechanism of the intermediate hosts is available. These gaps in our knowledge greatly limit us from taking effective measures to block the transmission from intermediate hosts. Practical experience tells us that biological control is superior to other means including physical or chemical control methods [17, 125]. However, this would require a long-term major project involving parasitologists and biologists (e.g., ecologists) working in collaboration. Intervention in the growth and reproduction of freshwater snails using biological control technology and the development of effective vaccines to prevent the infection of intermediate hosts (especially freshwater fish) with *C. sinensis* are feasible control strategies.

Third, due to deficiencies and imperfections of the food safety supervision network, the decreasing degree of freshwater fish infection degree by metacercariae [113] and limitations of the detection techniques used, a large number of positive, infected freshwater fish will remain in the market. In addition to fish meat digestion, researchers are experimenting with other sensitive detection methods, such as various PCR techniques and LAMP [116, 119, 120]. However, much work remains to explore and promote suitable detection methods that can be conveniently applied to market monitoring and field investigations.

Finally, the interaction between *C. sinensis* and its hosts, and the pathogenic mechanisms involved in clonorchiasis remain unclear. We need to further clarify the molecular mechanisms and immune cytokine network involved using omics and other feasible advanced technologies [69, 95] to screen for molecular markers that can be used for early diagnosis and monitoring disease progression, as well as drug targets.

## Conclusions

Due to the neglect and absence of systematic interventions, clonorchiasis remains prevalent worldwide, although some chemotherapy and control programmes have been implemented over several years in a few endemic areas [23, 126]. The wide distribution of intermediate hosts and reservoir hosts, human eating habits, the lack of the food safety supervision of freshwater fish, and relatively undeveloped techniques for detection and treatment are contributing to the prevalence of clonorchiasis. New and effective prevention and control strategies are urgently required. Importantly, the rapid development in *C. sinensis* omics (whole-genome, transcriptome and secretome) research has provided new opportunities for revealing the physiology, parasitism and pathogenesis of *C. sinensis*, for identifying target molecules that can be used in the development of new antiparasitic agents, and in screening appropriate diagnostic and vaccine candidates. New methods with higher sensitivity and specificity are being developed for detecting infection in human and intermediate hosts. Promising alternative drugs (e.g., tribendimidine) have proven more effective with fewer side effects than PZQ. Moreover, the new strategies of combining non-polluted fish culture with the use of fish vaccines might represent a viable alternative to block the transmission of *C. sinensis* and ensure the food safety of freshwater fish. We are confident that sustainable and innovative control strategies may eliminate clonorchiasis in the near future.

## Additional files

Additional file 1: Multilingual abstracts in the five official working languages of the United Nations. (PDF 524 kb) Additional file 2: Table S1. Infection rates of *C. sinensis* in freshwater snails in China (%). **Table S2.** Infection rates of *C. sinensis* in freshwater fish/shrimp in China (%). **Table S3.** Infection rate of *C. sinensis* in reservoir hosts in China (%). (DOC 201 kb)
