# Supplementary material for: Current status and perspectives of Clonorchis sinensis and clonorchiasis: epidemiology, pathogenesis, omics, prevention and control
Source: Infect Dis Poverty. 2016 Jul 6;5:71. doi: 10.1186/s40249-016-0166-1 (PMC4933995; doi:10.1186/s40249-016-0166-1)
Supplement: Additional file 1: — Multilingual abstracts in the five official working languages of the United Nations. (PDF 524 kb) [file 40249_2016_166_MOESM1_ESM.pdf]

Translation of the abstract into the five official working languages of the United Nations

**الوضع الحالي والرؤى لمتفرعات الخصية الصينية و داء متفرعات الخصية: الوبائية، الإمراض، اوميكس، الوقاية والسيطرة**

زي-لي تانغ، يان هوانغ، كسِن-بنغ يو

**خلاصة**

داء متفرعات الخصية الذي تسببه متفرعات الخصية الصينية (*C. sinensis*) هو أحد الأمراض الطفيلية المهمة التي تنتقل عن طريق الغذاء وهو أحد أكثر الأمراض حيوانية المصدر شيوعاً. في الوقت الحالي، يقدّر أنّ أكثر من 200 مليون شخص عرضة للإصابة بداء متفرعات الخصية، وأكثر من 15 مليون مصابين بالمرض في أنحاء العالم. إنّ عدوى متفرعات الخصية مرتبطة بقرب بسرطان الأقنية الصفراوية (CCA)، والتليّف وأمراض كبدية صفراوية بشرية اخرى؛ ولذلك، فإنّ داء متفرعات الخصية مشكلة صحة عامة خطيرة في المناطق المستوطنَة بالمرض. تعرِض هذه المقالة المعرفة الحالية المتعلقة بوبائية، وعبء المرض والعلاج لداء متفرعات الخصية، كما وتلخّص التقنيات المستخدمة للكشف عن عدوى متفرعات الخصية الصينية في البشر وفي المضيف الوسطي وتطوير اللقاحات ضد داء متفرعات الخصية. لقد تم جمع بيانات أجدد بخصوص إمراضية داء متفرعات الخصية، وجينوم (مَجين) وترانسكريبتوم (مجموع النواسخ) والسِكريتوم (مجموع الافرازات) لمتفرعات الخصية الصينية، موفرة رؤية للدراسات المستقبلية. ستساعد هذه التقدمات في المجال البحثي في تطوير استراتيجيات مبتكرة للوقاية والسيطرة على داء متفرعات الخصية.

Translated from English version into Arabic by Muther J. Alohmayed, through

TRANSLATORS
WITHOUT BORDERS

**华支睾吸虫及华支睾吸虫病的研究现状与展望：流行病学、发病机制、组学、预防及控制策略**

唐泽丽，黄艳，余新炳

**摘要：**华支睾吸虫病是由华支睾吸虫感染所引起的一种食源性寄生虫病，也是最常见的人畜共患病之一。目前，预计全球约 2 亿人受到华支睾吸虫感染的威胁，超过 1500 万感染者。在流行区，华支睾吸虫病是非常严峻的公共卫生问题。华支睾吸虫感染与人类的胆管癌、肝纤维化及其他肝胆管疾病的发生发展密切相关。本文对华支睾吸虫病的流行现状、疾病负担及诊断治疗等方面进行了综述，并对目前华支睾吸虫病诊断、疫苗及中间宿主（淡水螺、鱼）的华支睾吸虫感染检测技术的研究进展进行了总结。本综述还收集了华支睾吸虫病病理机制最新研究成果及华支睾吸虫基因组学、转录组学及蛋白质组学信息。这些研究及信息将有助于创新华支睾吸虫病的防治策略。

Translated from English version into Arabic by Tang Zeli and Huang Yan

**Situation et perspectives de *Clonorchis sinensis* et de la clonorchiase : épidémiologie, pathogenèse, économie, prévention et lutte**

Ze-Li Tang, Yan Huang, Xin-Bing Yu

**Résumé**

La clonorchiase, causée par *Clonorchis sinensis* (*C. sinensis*), est une importante maladie parasitaire d'origine alimentaire, et l'une des zoonoses les plus fréquentes. On estime actuellement que plus de 200 millions de personnes courent un risque d'infestation par *C. sinensis* et que plus de 15 millions sont infestées dans le monde. En raison du lien étroit établi entre l'infestation par *C. sinensis* et le cholangiocarcinome, la fibrose hépatique et d'autres maladies hépatobiliaires chez l'homme, la clonorchiase constitue un sérieux problème de santé publique dans les régions d'endémie. Notre article passe en revue les connaissances actuelles sur l'épidémiologie, le fardeau de la maladie et le traitement de la clonorchiase et résume les techniques de détection de l'infestation par *C. sinensis* chez ses hôtes intermédiaires et l'homme, ainsi que le développement d'un vaccin contre la clonorchiase. Des données récentes concernant la pathogenèse de la clonorchiase et le génome, le transcriptome et le sécrétome de *C. sinensis* ont été recueillies et constitueront le matériau d'études futures. Ces progrès de la recherche aideront à développer de nouvelles stratégies pour la prévention et la lutte contre la clonorchiase.

Translated from English version into French by Suzanne Assenat, through

TRANSLATORS WITHOUT BORDERS

**Гельминтоз, вызываемый *Китайской двуусткой* и клонорхозы - современное состояние проблемы и перспективы ее решения: эпидемиология, патогенез, -омики*, профилактика и контроль заболеваемости**

Цзэ Ли Тан, Янь Хуан, Синь Бин Юй

**Резюме**

Клонорхоз – это паразитарное заболевание пищевого происхождения, вызываемое *Китайской двуусткой* (*C. sinensis*) и один из самых распространённых зоонозов** . В настоящее время предполагается, что более 200 миллионов человек подвержены риску заражения клонорхозом и свыше 15 миллионов человек были инфицированы по всему миру. Клонорхоз тесно связан с холангиокарциномой (CCA), муковисцидозом и другими заболеваниями печени и желчных путей, поэтому холангиокарцинома представляет собой серьезную проблему общественного здравоохранения в эндемических районах. В этой статье рассматриваются имеющиеся эпидемиологические данные в отношении бремени болезни, лечения клонорхоза, а также содержится краткая информация о методах выявления клонорхоза в организме человека и в организме промежуточных хозяев, а также информация о разработке вакцин против клонорхоза. Получены новые данные о патогенезе клонорхоза, а также о геноме, транскриптоме и секретоме (секретируемом протеоме) возбудителя заболевания – *Китайская двуустка* (C. sinensis), которые открывают перспективы для будущих исследований. Эти достижения в научно-исследовательской деятельности будут способствовать разработке новаторских стратегий по предупреждению и сдерживанию распространения клонорхоза.

Translated from English version into Russian by Tatsiana Mankevich, through

**Estado actual y perspectivas del *Clonorchis sinensis* y la Clonorquiasis: epidemiología, patogénesis, ómicas, prevención y control**

Ze-Li Tang, Yan Huang, Xin-Bing Yu

**Resumen**

La clonorquiasis, producida por el *Clonorchis sinensis* (*C. Sinensis)*, es una importante enfermedad parasitaria transmitida por alimentos y una de las zoonosis más comunes. En la actualidad, se estima que más de 200 millones de personas corren riesgo de infección por *C. sinensis* y más de 15 millones están infectadas en el mundo entero. La infección por *C. sinensis* está íntimamente relacionada con el colangiocarcinoma (CCA), la fibrosis y otras enfermedades hepatobiliares humanas. Por lo tanto, la clonorquiasis es un serio problema de salud pública en las zonas endémicas. El presente artículo repasa los conocimientos actuales en cuanto a la epidemiología, carga de la enfermedad y tratamiento de la clonorquiasis y hace un resumen de las técnicas para la detección de la infección por *C. sinensis* en el ser humano y huéspedes intermedios y el desarrollo de la vacuna contra la clonorquiasis. Se recolectó información más reciente sobre a la patogénesis de la clonorquiasis y el genoma, transcriptoma y secretoma de *C. sinensis* lo que ofrece perspectivas para futuros estudios. Estos avances en la investigación ayudarán al desarrollo de estrategias innovadoras para la prevención y control de la clonorquiasis.

Translated from English version into Spanish by Maria Alejandra Aguada, through
