# Supplementary material for: Current status and perspectives of Clonorchis sinensis and clonorchiasis: epidemiology, pathogenesis, omics, prevention and control
Source: Infect Dis Poverty. 2016 Jul 6;5:71. doi: 10.1186/s40249-016-0166-1 (PMC4933995; doi:10.1186/s40249-016-0166-1)
Supplement: Additional file 2: Table S1. — Infection rates of C. sinensis in freshwater snails in China (%). Table S2. Infection rates of C. sinensis in freshwater fish/shrimp in China (%). Table S3. Infection rate of C. sinensis in reservoir hosts in China (%). (DOC 201 kb) [file 40249_2016_166_MOESM2_ESM.doc]

**Table S1 Infection rates of *C. sinensis*** in freshwater snails in China (%)

| **P/A** | **Area** | **Species of freshwater snails** | | | | **Source** | **Years** |
| --- | --- | --- | --- | --- | --- | --- | --- |
|  |  | **1** | **2** | **3** | **4** |  |  |
| Guangdong | Shunde | 1.64 (1/61) | 5.48 (4/73) | 0 (0/1) |  | Fishponds | 2008-2009 [1] |
|  | Shaoguan | 2.8 (31 /11065) | 0.8 (3/340) |  |  | Fishponds, ditches | 2002-2006 [2] |
|  | Shaoguan | 2.89 (32/11083) | 0.91 (4/440) |  |  | Fishponds, ditches | 2002-2008 [3] |
|  | Shenzhen | 0.96 (3/310) | 0 (0/30) | 0 (0/30) | 1.25 (2/160) | Fresh lake | 2010 [4] |
| Guangxi | Wuming | 0.18 (4/2218) | 1.07 (65/6072) | 1.03 (59/5733) |  | Fishponds, ditches | 1990-2003 [5] |
|  | Binyang | 0.44 (3/678) | 2.52 (19/754) | 1.28 (8/628) |  | Fishponds, ditches | 2001-2006 [6] |
| Heilongjiang | Eastern | 0.13 (2/1579) |  |  |  | River | 2002 [7] |
| Liaoning | 5 cities | 5.13 (22/413) |  |  |  | Rivers | 2007 [8] |
| Hunan | Southern | 17.4 (29/67) | 7.4 (2/27) |  |  | Fishponds, ditches | 2006-2010 [9] |
| Jiangsu | Xinyi | 1.89 (8/435) | 0.35 (2/572) |  |  | Wild | 2006 [10] |
| Anhui | Huainan | 0.25 (5/2000, freshwater snails) | | | | Waters | 2004 [11] |

**Notes: 1 *Parafossarulus striatulus*; 2 *Alocinma longicornis*; 3 *Bithynia fuchsianus*; 4 *Parafossarulus sinensis*.**

**Table S2 Infection rates of *C. sinensis*** in freshwater fish/shrimp in China (%)

| **P/A/M** | **Area** | **Species of freshwater fishes/ shrimps** | | | | | | | | | | | **Source** | **Years** |
| --- | --- | --- | --- | --- | --- | --- | --- | --- | --- | --- | --- | --- | --- | --- |
|  |  | **1** | **2** | **3** | **4** | **5** | **6** | **7** | **8** | **9** | **10** | **Others** |  |  |
| Guangdong | Guangzhou |  | 10 | 5.6 |  |  | 32 |  | 50 |  |  |  | Markets, hatcheries | 2005 [12] |
|  | Guangzhou |  | 13.33 |  |  |  |  |  |  |  |  | 11 (6.67) | Caterings | 2006 [13] |
|  | Guangzhou |  | 4 | 0 | 6 |  | 0 |  |  |  |  |  | Markets | 2007 [14] |
|  | Shunde | 25 | 57.1 | 25 | 16.7 |  | 0 | 0 |  |  |  |  | Markets, hatcheries | 2005 [12] |
|  | Sanshui |  | 100 |  |  |  | 52.1 |  |  |  |  | 11 (7.1) | Fishponds | 2006 [15] |
|  | zhongshan | 81.3 | 75.7 |  |  |  |  |  |  |  |  |  | Fishponds | 2006 [15] |
|  | Shaoguan | 84.3 | 9 |  |  |  |  | 5 | 25 |  |  |  | Fishponds | 2002-2006 [2] |
|  | Shaoguan | 81.43 | 5.4 |  |  |  |  | 3.6 | 9.6 |  |  |  | Fishponds | 2002-2008 [3] |
|  | Jiangmen |  | 50 | 9.1 | 12.8 |  | 3.6 |  |  |  |  |  |  | 2012 [16] |
|  | Jiangmen |  | 47.93 |  | 15.7 |  |  |  |  |  |  |  | Fishponds | 2009 [17] |
|  | Jiangmen | 55.6 | 5.1 |  |  | 22.6 |  |  |  |  |  |  | Eateries | 2008-2009 [18] |
|  | Zhaoqing |  | 20 | 0 | 12.8 | 17.9 | 0 | 0 |  |  |  |  | Markets | 2006 [19] |
|  | Zhaoqing |  | 48.8 |  | 37.5 |  |  |  |  |  |  |  | Fishponds | 2009 [20] |
|  | Shenzhen | 26 | 15 | 8 | 7 | 8 | 16 |  |  |  |  |  | Fresh lake | 2010 [4] |
|  | Shenzhen |  | 40.74 | 14.50 | 22.85 | 22.5 | 26 |  |  |  |  | 12 (16.6) |  | 2005 [21] |
|  | Shenzhen |  | 30.77 | 16.67 | 14.29 | 13.33 |  | 0 |  |  |  | 12 (0), 13 (9.52) | Sales locations | 2007 [22] |
|  | Shenzhen |  | 8.33 | 7.69 | 8.33 | 0 |  | 20 |  |  |  | 12 (18.18), 13 (28.57) | Sales locations | 2008 [22] |
|  | Shenzhen |  | 40.2 | 19.6 | 17 | 19.4 | 18.6 |  |  |  |  | 12 (16) | Markets | 2009 [23] |
|  | 4 cities |  | 47.31 | 28.58 | 23.29 |  | 6.76 | 45.5 |  |  |  |  | Fishponds | 2008-2009 [1] |
|  | Huizhou |  | 11.9 |  | 1.7 |  |  |  |  |  |  |  | Fishponds, market | 2008 [24] |
|  | Dongguan |  | 82.7 | 0 |  |  |  |  |  |  |  |  | Fishponds | 2008 [25] |
| Guangxi | Nanning |  | 0 | 10 | 5 | 23.3 | 0 | 5 |  |  |  |  | Markets | 2006 [26] |
|  | Heng | 34.2 |  |  |  |  | 0 |  | 11.63 |  |  |  | Reservoir | 2011 [27] |
|  | Heng |  | 9.5 | 3.7 |  | 12 |  | 7.7 |  |  |  | 11 (0) | Markets | 2009 [28] |
|  | Heng | 28.8 |  |  |  |  |  |  |  |  |  |  | Reservoir | 2012 [29] |
|  | Liujiang River | 21.5 | 12.7 | 8.3 | 8.4 | 8.1 | 8.6 | 10.7 |  | 5.9 | 4.5 |  | River | 2008-2009 [30] |
|  | Wu Ming | 22.11 | 10.53 | 20 | 40 | 16.67 | 0 | 5.97 |  |  |  |  | Fishponds, ditches, markets | 1990-2003 [5] |
|  | Bingyang | 8.46 (Freshwater fishes) | | | | | | | | | | | Fishponds, ditches | 2001-2006 [6] |
| Heilongjiang | Qigihar | 41.6 |  | 45.9 |  |  |  |  | 60 | 82.7 |  |  | Rivers | 2013 [31] |
|  | Qigihar | 39.8 |  | 36.8 |  | 8.33 |  |  |  |  | 2.3 |  | Markets | 2012 [32] |
|  | Eastern | 100 |  | 20.3 |  |  |  |  | 80.1 | 60.0 | 20.0 |  | Rivers | 2002 [7] |
| Jilin | Changchun |  |  | 0 |  | 5.26 |  | 0 |  |  |  | 14 (0) | Markets | 2008 [33] |
|  | Changchun | 33.3 | 8.5 | 10 |  |  |  |  |  | 29.6 |  |  | Markets | 2009 [34] |
| Liaoning | 5 cities | 54.36 | 28.57 | 2.75 |  | 0 |  |  |  |  | 50 | 15 (13.21), 16 (18.78), 17 (7.56) | Markets, rivers | 2007 [8] |
|  | western | 82.8 |  | 48.1 |  |  |  |  |  |  |  | 14 (6.6), 18 (59.3), 19 (1.5), 20 (70.9) | Rivers | 2009 [35] |
|  | Jinzhou | 98.41 | 10.42 | 44.90 | 21.21 | 20.75 |  | 8.57 |  |  |  | 14 (12.5), 18 (61.29), 19 (1.54), 20 (74.51) | Markets | 2009 [36] |
|  | Dalian | 77.8 |  |  |  | 5 |  | 0 | 12.5 |  |  | 21 (95.8), 22 (13.3) | Waters, markets | 2008 [37] |
|  | Dalian | 48 |  | 0 |  | 0 |  |  | 0 |  | 0 | 16 (7.58), 21 (100), 22 (14.58) | River, markets | 2008 [38] |
| Hunan | Southern |  | 0 | 69.2 |  | 5.3 |  |  |  |  |  |  | Fishponds, Markets | 2006 [9] |
|  | Southern |  |  | 2.3 |  |  |  |  |  |  |  |  | Fishponds, Markets | 2009 [9] |
|  | Yongzhou |  | 9.38 | 14.63 | 8.33 | 7.89 |  | 4.76 |  |  |  | 23 (11.11) | Restaurants | 2006 [39] |
| Chongqing | Tongnan | 25 |  | 0 |  |  |  |  |  |  |  | 24 (6.9) | River | 2006 [40] |
| Jiangsu | Nangtong | 20 (Freshwater fishes) | | | | | | | | | | | Markets | 2012 [41] |
|  | Xinyi | 57.1 | 0 |  |  | 0 |  | 0 |  |  |  | 21 (6.4) | Wild | 2006 [10] |
|  | Northern | 7.92 |  | 2.27 |  |  |  |  | 0 |  |  | 21 (3.45), 24 (4.05) |  | 2009 [42] |
|  | Jinghu | 6.72 (more than three pieces of freshwater fishes) | | | | | | | | | | | Wild | 2009 [43] |
|  | Suzhou | 72.1 |  | 11.1 |  |  |  |  | 19 |  | 4 | 11 (2), 23 (6.7), 24 (3.2) | Markets | 2004 [44] |
| Anhui | Huainan | 33.3 | 2.5 | 15.83 |  | 30 |  | 8.3 |  |  |  | 11 (5.83), 23 (6.66), 25 (7.5) | Waters, Markets | 2004 [11] |
|  | Huainan | 31.7 |  | 6.5 |  | 6.2 | 2.6 | 5.3 |  |  |  | 23 (0) | Markets | 2003 [45] |
| Tianjin |  | 6.44 |  |  |  |  |  |  |  |  |  |  | Rivers | 2006 [46] |
| Hubei | Jingzhou | 33.33 |  | 0 |  | 0 |  | 25 | 25 |  |  | 21 (23.33), 23 (0) | Markets | 2006 [47] |
|  | Xianning | 75.13 |  | 9.94 | 15.11 |  |  |  |  |  |  | 26 (39.13) | Markets | 2011 [48] |
| Hebei | Tangshan | 83.41 |  |  | 2.2 | 0 |  |  |  |  |  | 16 (40), 27 (12.82) | Markets | 2009 [49] |
| Zhejiang | Tonglu | 7.34 |  | 0 |  |  |  |  | 0 |  |  | 28 (3.32), 29 (5.56) | Rivers | 2012 [50] |
|  | Western | 20.6 |  | 5.2 | 9.5 | 0 |  |  | 13.3 |  | 12.9 | 11 (3), 18 (10.8), 16 (7.1), 29 (1.3), 30 (15.5), 31 (3.8), 32 (12) | Rivers | 2012 [51] |
|  | Jinhua | 17.65 |  | 5.34 | 9.52 |  |  |  | 17.7 |  | 13.3 | 11 (2.96), 16 (10.5), 18 (10.8), 29 (2.94), 31 (4.76), 33 (12) | Wild | 2012-2013 [52] |
|  | Northern | 19.29 |  |  |  |  |  |  |  |  |  | 21 (35.53) | Rivers | 2010 [53] |
| Shanghai |  | 7.83 |  | 0 |  | 0 |  |  | 1.35 |  |  | 21 (0), 16 (2.3) | Markets | 2005-2010 [54] |

**Notes:** **1 *Pseudorasbora parva*; 2** ***Ctenopharyngodon idellus*; 3 *Carassius auratus*; 4 *Hypophthalmichthys nobilis*; 5 *Cyprinus carpio*; 6 *Cirrhinus molitorella*; 7 *Hypophthalmichthys molitrix*; 8 *Cyprinidae Rhodeus*; 9 *Saurogobio dabryi*;** **10 *Misgurnus anguillicaudatus*; 11 shrimp; 12 *Channa argus*; 13 *Oreochromis mossambicus*; 14 *Parasilurus asotus*; 15 *Pelteobagrus fulvidraco*; 16** ***Hemicculter Leuciclus*; 17 *Gobio gobio maacrocephalus Mori*; 18 *Erythroculter ilishaeformis*; 19 *Phoxinus lagowskii Dybowski*; 20 *Perccottus glenii*; 21** ***Abbottina rivularis*; 22 *Ctenogobius giurinus*; 23 *Parabramis pekinensis*; 24 *Hemiculterella sauvagei Warpachowsky*; 25 *Mylopharyngodon piceus*; 26 *Megalobrama amblycephala*; 27 *Sebastiscus marmoratus*; 28 *Gambusia affinis*; 29 *Acrossocheilus fasciatus*; 30 *Opsariichthys bidens Gunther*; 31 *Distoechodon tumirostris Peters*; 32 *Heros managuense*; 33 *Cichlidae*.**

**Table S3 Infection rate of *C. sinensis* in reservoir hosts in China (%)**

| **P/A** | **Area** | **Cats** | **Dogs** | **Pigs** | **Others** | **Years** |
| --- | --- | --- | --- | --- | --- | --- |
| Guangdong | Pearl River Delta | 41.7 (214/516) |  |  |  | 2010-2012 [55] |
|  | Jiangmen | 45.45 (10/22) | 36.84 (7/19) | 0 |  | 2012 [15] |
|  | Shaoguan | 51.52 (17/33) | 18.6 (16/86) | 0/58 | Cattle (0/27) | 2002-2008 [3] |
|  | Shaoguan | 65.22 (15/23) | 17.45 (14/78) | 0/49 | Cattle (0/17), voles (0/13) | 2002-2006 [2] |
| Guangxi | Shanglin |  | 53.66 (22/41) |  |  | 2008 [56] |
|  | Wuming | 40 (2/5) | 16.67 (1/6) | 0/52 | Voles (0/63) | 1990-2003 [5] |
|  | Binyang | 86.33 (21/26) | 36.36 (12/35) |  | Ducks (0/67), voles (0/300) | 2001-2006 [6] |
| Heilongjiang | Eastern | 93.75 (30/32) | 33.33 (7/21) | 0/3 | Rabbits (100%, 2/2) | 2002 [7] |
|  | Qigihar | 6.67 (1/15) | 8.57 (3/35) |  |  | 2013-2014 [57] |
| Jilin | Zhenlai |  | 27.3 (3/11) |  |  | 2006-2009 [58] |
| Liaoning | Dalian | 0/10 | 5.56 (2/36) | 0/6 |  | 2008 [33] |
|  | Pulandian |  |  | 4 (2/50) |  | 2015 [59] |
| Hunan | Southern | 0/1 | 85 (6/7) |  | Rattus norvegicus (0/6) | 2006-2009 [9] |
|  | Yongzhou | 0/1 | 100 (3/3) |  | Rattus norvegicus (0/6) | 2006 [60] |
| Hubei | Gongan | 70 (7/10) |  | 26.3 (5/19) | Cattle (0/3), chicken (0/10) | 2006 [61] |
| Jiangsu | Xinyi | 73 (22/30) | 60 (6/10) |  |  | 2006 [10] |
|  | Northern | 20.75 (11/53) | |  |  | 2009 [38] |
| Fujian | Fuzhou |  | 2.09 (11/526) |  |  | 2009-2010 [62] |
| Shandong | Weishan | 38.46 (15/39) |  |  |  | 2002 [63] |
| Gansu | Lanzhou | 7.02 (4/57) | 1.56 (4/256) |  |  | 2008-2009 [64] |
| Zhejiang |  |  | 1.13 (4/353) |  |  | 2014 [65] |

**Supplementary** **References**

1. Zhang XC, Pei FQ, Zhang QM, Lin RX, Huang ShY, Wang JL, et al. Current status of environmental sanitation and *Clonorchis sinensis* intermediate host infection of freshwater aquaculture in partial areas of Guangdong Province. South Chin J Prev Med. 2010;36(3):9-13.

2. Zou XH, He LJ, Luo JP, Chen LG, Pan D. Survey of *Clonorchis sinensis* in Zhenjiang Dstrict of Shaoguan City. Chin Trop Med. 2007;7(11):2089-90.

3. [Cui WJ, Zou XH, He LJ, Chen LG, Luo JP, Pan D. Survey of infectious status of *Clonochis sinensis* in urban area of Shaoguan City in 2002-2008.](http://114.255.123.115/zh/detail.do?ui=2011247066) Chin Trop Med. 2009;9(10):2017-8.

4. [Peng ZQ, Geng YJ, Huang ND, Gao ST, Li XH, Zhang RL. Evaluation of effectiveness of integrative intervention on *Clonochis sinensis* infection in a village of Shenzhen City.](http://114.255.123.115/zh/detail.do?ui=2010528256) Chin Trop Med. 2010;10(4):413-4,430.

5. Su LJ, Lu ZB, Lu L, Liang MQ, Wei SZ. [Survey on the Prevalence of *Clonorchis sinensis* in Wuming, Guangxi](http://114.255.123.115/zh/detail.do?ui=2006278298). J Trop Med. 2006;6(9):1017-9.

6. [Liang H, Qu JX, Li W, Huang GW, Ma Z. Epidemiological survey of prevalence of clonorchiasis in Binyang County](http://114.255.123.115/zh/detail.do?ui=2010490590). Chin Trop Med. 2009;9(4):709-10.

7. Cai LS, Xin H, Chen G, Wen GZ. Epidemic investigation of clonorchiasis in the eastern area of Heilongjiang province. Heilongjiang Med & Pharm. 2002;25(6):14-15.

8. Li Y, Tao YC, Teng C, Yao WQ. [Prevalence of clonorchiasis in endemic areas of Liaoning province](http://114.255.123.115/zh/detail.do?ui=2013278521). Chin J Public Health. 2013;29(2):261-3.

9. Chen PH, Duan JH, Tang Y, Chen WH, Tang XY, Wu YJ, etal. Study on risk factors and comprehensive control and prevention mode of *Clonorchis sinensis* infection in high-risk areas in Hunan Province. Pract Prev Med. 2012;19(3):344-8.

10. Zhang ZC, Suo GH, Lou PA, Li HM. Investigation of *Clonorchis sinensis* intermediate and reservoir hosts infection in Xinyi city. Chin J School Doctor. 2007;21(4):426.

11. Guo J, Wang KX. Investigation on intermediate host infection of *Clonorchis sinensis* in Huainan area. Chin J Public Health. 2006;22(8):968-9.

12. Huang J, Liang YJ, Lin CK, Huang ZH, Feng SY, Chen JJ, et al. A Survey on the infection of freshwater fish by *Clonorchis sinensis* in Shunde, Panyu, Qingyuan, Zengcheng and Huadu, Guangdong province. J Trop Med. 2006;6(3):331-3.

13. Zhang YL, Feng YJ, He JY, Liu XN, Qu JJ, Deng XB. Foodborne parasitic infection in Guangzhou area-prevention and treatment strategies. J Trop Med. 2006;6:1282-4.

14. Lu XH, Yu WQ, Liang XY, Li QX, Wu MH, Zeng WT, et al. Investigation of *Clonorchis sinensis* metacercaria infection in freshwater fish in meat market of Guangzhou city. 2007;7(12):150-1.

15. Liu XD, Zhu CH, Li YL, Lao MX, Zhu MC, Li ZC, et al. Investigation on infection of *Clonorchis sinensis* in freshwater fishes and shrimps from Sanshui and Zhongshan of Guangdong province. Prac Prev Med. 2007;14(1):84-5.

16. Liu YH, Yi BK, Zheng NC, Liang BN, Li FL, Tan XF, et al. [Investigation of the epidemiological characteristic of clonorchiasis in Jiangmen Pengjiang District](http://114.255.123.115/zh/detail.do?ui=2013218317). J Trop Med. 2012;12(11): 1385-7.

17. Feng DN, Liang HY, Zhu YQ, Li X, Li WJ, Zhang XQ, et al. [Investigation of *Clonorchis sinensis* infection in freshwater fishes in Jiangmen](http://114.255.123.115/zh/detail.do?ui=2010506966). Parasito & Infect Dis. 2010;8(1):4-6.

18. Huang XH, Li ZQ, Zhang XH, Liu DX, Liang SL, Huang ZX, et al. Survey of *Clonorchis sinensis* infection infreshwater fish at eatery in Yangshan County. J Trop Med. 2010;10(7):896-7.

19. Gao XX, Qu Y, Li WW, Guo YL, Wu L. Investigation of *Clonorchis sinensis* infection of the intermediate host and the epidemiology of *Clonorchiasis Sinensis* in Zhaoqing City. J Trop Med. 2007;7(6):608-9,591.

20. Chen XY, Lu JY, Qiu ZC, Zou YQ, Wang RS, Xu J, et al. [Investigation of infection with *Clonorchis Sinensis* in freshwater fishes from Zhaoqing City](http://114.255.123.115/zh/detail.do?ui=2010538650). J Med Pest Control. 2010;(1): 1-2.

21. Huang DN, Gao ST, Geng YJ, Huang W, Li H, et al. Investigation of *Clonorchis sinensis* infection in freshwater fish in Shenzhen city. J Trop Med. 2006;6:42-4.

22. Wang Z, Huang W, Pan LB, Tan W. Investigation on the pollution of aquatic products in Shenzhen from 2006 to 2008. Chin J Food Hyg. 2010;22:165-7.

23. Huang FY, Zhang QW, Geng YJ, Huang DN, Li XH, Gao ST, et al. Investigation into infection route and factors associated with *Clonorchis sinensis* infection in freshwater fish in Shenzhen city. Chin Trop Med. 2009;9:114-6.

24. Che YC, Chen JJ, Lan YF, Qiu SC, Pan JX, Zhang DM, et al. Investigation on the infection of encysted metacercariae of *Clonorchis sinensis* in two kinds of freshwater fish from Huizhou City in 2008. Prev Med Trib.2009;15(3):212-3.

25. Liang BF, Zhong YF, Cai YT, Xie X, Liang LY, Yang HB, et al. Survey of infection status of *Clonorchis sinensis* in freshwater fish in Dongguan city of Guangdong province. Chin Trop Med. 2009;9(3):521-2.

26. Ma YX, Gao Y, Ma MG, Tan XL, Chen C, Hu WQ. [Investigation on the commercial freshwater fish infection with encysted Metacercaria of *Clonorchis sinensis* in Nanning](http://114.255.123.115/zh/detail.do?ui=2007183391). Applied Prev Med. 2007;13(2):80-2.

27. Zhu TJ, Li HM, Wang JJ, Zhu HH, Zang W. [Infection status of freshwater fishes with metacercariae of fish born trematode in Heng County Guangxi](http://114.255.123.115/zh/detail.do?ui=2012543277). Int J Med Parasit Dis. 2012;39(3):167-170.

28. Huang QM, Lu Y. Infection investigation of encysted metacercaria of *Clonorchis Sinensis* in freshwater fishes and shrimps at County Heng Trade market in Guangxi. Henan J Prev Med. 2009;20(5):349-50.

29. Li HM, Zhu HH, Zhu TJ, Zang W, Chen YD. [Comparison on two methods for detecting metacercariae of *Clonorchis sinensis*](http://114.255.123.115/zh/detail.do?ui=2012543281). Int J Med Parasit Dis. 2012;39(3):152-4.

30. Shen HG, Zhou ZZ, He QB, Mo HY, Tao J, Huang SQ. Investigation on the fish infected metacercaria of *Clonorchis Sinensis* in the Liujiang river. Mod Prev Med. 2010;37:1549-52.

31. Liu JX, Sun YH, Zhang H, Li CP. Prevalence of metacercariae of *Clonorchis sinensis* in wild freshwater fishes from Nenjiang River around Qiqihaer City. Zhongguo Ji Sheng Chong Xue Yu Ji Sheng Chong Bing Za Zhi. 2014;32(4):292-4.

32. Sun YH, Zhang H, Liu JX. Investigation of *Clonorchis sinensis* metacercaria infection in freshwater fish in integrated markets of Qiqihar city. J Qiqihar Univ Med 2014;35:860-1.

33. Liu L, Dong JH, Geng ZH. Investigation of *Clonorchis sinensis* infection in freshwater fish in the Changchun City integrated market. J Pathog Biol. 2009;4(6):2,404.

34. Ye CY, Wang ZJ, Wu XP, Li YX, Yang XY, Wang XL, et al. [Investigation on the commercial freshwater fishes that infection with metacercaria of *Clonorchis sinensis* in Baicheng region.](http://114.255.123.115/zh/detail.do?ui=2010480079) Mod Prev Med. 2009;36(1):127,130.

35. Liu XG, Liu G, Zhang WW. Infection status of wild freshwater fish with Metacercariae of *Clonorchis sinensis* in west Liaoning. Zhongguo Ji Sheng Chong Xue Yu Ji Sheng Chong Bing Za Zhi. 2011;29(2):157-8.

36. Liu G, Yu JL, Xu P, Liu XG. Survey of infectious status of fresh water fish with *Opisthorchis sinensis* in Jinzhou city. Chin J Zoon. 2010;26:714-5.

37. Chen FY, Zheng LL, Yao W, Ao JK, Qin YH, Ren YX, et al. [Epidemiological investigation of *Clonorchiasis sinensis* in Dalian area](http://114.255.123.115/zh/detail.do?ui=2008172162). J Pathog Biol. 2008;3(3):221-222,3.

38. Mei D, Chen FY, Liu DH, Yao W, Bo YZ, Zhang B, et al. Investigation on the infection of *Clonorchis sinensis* in intermediate host and persons in rural areas of Dalian. J Pathog Biol. 2008;3(3):4,230.

39. Wang HP, Tang SL, Li L. Survey of infectious status of fresh water fish with *Clonorchis sinensis* in Yongzhou City,Hunan Province. Chin Trop Med. 2006; 6(4):597-8.

40. Jiang SG, Yuan NL, Wu CG, Wang JG, Zhou ZL, Li JG, et al. Surveillance of *Clonorchiasis Sinensis* in Tongnan County in 2006. J Trop Med. 2007;7(6):603-5,602.

41. Zhang ZJ, Zhang WB, Zhao RM, Shen MX, Jiang WC, Jin F, et al. The status of common fishes infection with *Clonorchis sinensis* and *Anisakis marina* in Nantong city. Chin J Prev Med. 2013;47(7):669.

42. [Shen MX, Jin XL, Li J, Xu XZ, Cao HY, Jiang WC, et al. Investigation on epidemic status of clonorchiasis in Northern Jiangsu Province](http://114.255.123.115/zh/detail.do?ui=2011195759). Chin J Schisto Control. 2010;(5):468-47.

43. Sun DK, Zhang CP, Li Q, Li SM, Yang WZ, Jia CY, et al. Epidemic situation of *Clonorchis sinensis* in Jinhu County. Chin J Schisto Control. 2010;22(4):381-3.

44. Yuan HX, Gao S, He YP, Wang P, Su ZH. Investigation on the infection of *Clonorchis sinensis* metacercaria in freshwater fishes and shrimps from markets of Suzhou area. Chin J Parasit Dis Con. 2005;18(5):399.

45. Cai R, Li CP, Wang J, Zhan XD, Li M, Ke XF. Survey of infectious status of fresh water fish with *Opisthorchis sinensis* in Huainan area. Chin J Parasitol Parasit Dis. 2005;23:39.

46. Di BH, Li X, Xu YD. Investigation of *Pseudorasbora parva* infected with *Clonorchis sinensis* in the main rivers in Tianjin city. Chin J Epidemiol. 2007;28(8):809.

47. Ding YL, Xia P. Survey and analysis of 7 species of freshwater fish infection with metacercariae of *Clonorchis sinensis* in Jingzhou City. J Yangtze Univ ( Nat Sci Edit) Med V. 2007;4(3):233-4.

48. Luo C, Chen XG, Tan JH, Zhao GH. Investigation of infection with *Clonorchis sinensis* in freshwater fish from Xianning City. J Pathog Biol. 2011;6(9):687-8.

49. Liu JW, Liu XQ, Niu LP, Li CH, Li QX, Xie Y, et al. Study of the infection of metacercariae of *Clonorchis sinensis* in freshwater fish in Tangshan. J Pathog Biol. 2010;5(3):207-9,4.

50. Wu BY, Fang SY, Tong YQ. Survey the situation of *Clonorchis sinensis* infection in freshwater fish in Tonglu county. Zhejiang Prev Med. 2013;25(12):49-50.

51. Chen HL, Wang DB, Luo XJ, Chen PF, Yao LN, Xia SR, et al. Prevalence of *Clonorchis sinensis* infection in Western Area of Zhejiang Province. Dis Surveil. 2013;(6):499-502.

52. Yu XT, Luo XJ, Chen PF, Zhang GM, Chen ZX, Wang XH, et al.[An investigation on the status of wild freshwater fish and shrimp infected with metacercaria of *clonorchis sinensis* in Jinhua City](http://114.255.123.115/zh/detail.do?ui=2015736228). Zhejiang Prev Med. 2015; 27(8):772-4.

53. Yang TT, Yao LN, Yao SR, Ruan W, Chen HL, Xia SR, et al. Assessment of *Clonorchis Sinensis* epidemic trends and its natural epidemic focus in northern area of Zhejiang Province. Zhejiang Prev Med. 2011;23(5):17-8,22.

54. Zhang XP, Jiang SF, Hong GB, Fu YH, He YY, Ma XJ, et al. Investigation on food contamination with parasites in Shanghai market. Chin J Schisto Control. 2012;24(4):404-9.

55. Wang GY, Wang M, Guan XY, Tai SS, Liu GQ, Gao XL, et al. A study of natural infection with *Clonorchis sinensis* in domestic cats in the Pearl River Delta region of Guangzhou and creation of an animal model of clonorchiasis using guinea pigs. J Pathog Biol. 2013;8(11):966-8,985.

56. Ruan TQ, Zhang HM, Tan YG, Huang FM, Lin R, Ou YY, et al. Survey on the transmission potential of clonorchiasis by dogs in Shanglin County, Guangxi. J Pathog Biol. 2008;3(10):773-4.

57. Sun YH, Liu JX, Sun Y, Yao SJ, Guo J, Zhang J, et al. Investigation on *Clonorchis sinensis* infection in dogs and cats in Nenjiang River basin Qiqihaer City. Int J Med Parasit Dis. 2015; 42(2):99-100,103.

58. Yang XY, Li YX, Bai YS, Shi ZS, Wang LB, Li XS, et al. Epidemiological investigation on *Clonorchis sinensis* infection in Nenjiang river basin of Zhenlai county, Jilin province by modified kato-Katz thick smear method. Chin J Lab Diagn. 2011;15:1721-2.

59. Xiao B. A report about diagnosis and treatment of pigs clonorchiasis. Chin J Anim Husb Veter Med. 2015; (11):80.

60. Duan JH, Tang XY, Wang QZ, Tang Y, Zhang ZS, Li ZX, et al. Epidemiological survey on *Clonorchiasis sinensis* in an endemic area of South Hunan Province. Zhongguo Ji Sheng Chong Xue Yu Ji Sheng Chong Bing Za Zhi. 2009;27(6):467-71.

61. Zou SC, Gong AC, Wang YA. Epidemiological investigation of *Clonorchiasis sinensis* in Gongan County of Hubei province. Chin J Clin Med Res. 2006;12(11):1535-6.

62. Lin XQ, Yu XX. Survey of gastrointestinal parastites in dogs in Fuzhou. Fujian Xu Mu Shou Yi. 2015;37:7-10.

63. Miu F, Yan XZ, Liu X. [Study on influence of *Clonorchis sinensis*/Clonorchiasis epidemic caused by ecological environment changes inWeishan lake area](http://114.255.123.115/zh/detail.do?ui=2005770513). Chin J Public Health. 2005, 21(10): 1191-2.

64. Lu WY, Ha L, Cao LP. Investigation into intestinal parasites infections in clinic dogs and cats in Lanzhou city. Chin J Anim Infect Dis. 2010;18:72-4.

65. Pan C, Yang Y, Chen XQ, Du AF. [Development and primary application of an indirect ELISA for detecting *Clonorchis sinensis*](http://114.255.123.115/zh/detail.do?ui=2016166886). Chin J Veter Science. [2015;35(11)](javascript:searchLink('','true','zh','@中国兽医学报@%5B刊名%5D   AND @2015@%5B年%5D   AND @11@%5B期%5D');): 1792-8.
